# Transgenic Resistance Confers Effective Field Level Control of Bacterial Spot Disease in Tomato

**DOI:** 10.1371/journal.pone.0042036

**Published:** 2012-08-01

**Authors:** Diana M. Horvath, Robert E. Stall, Jeffrey B. Jones, Michael H. Pauly, Gary E. Vallad, Doug Dahlbeck, Brian J. Staskawicz, John W. Scott

**Affiliations:** 1 Two Blades Foundation, Evanston, Illinois, United States of America; 2 Plant Pathology Department, University of Florida, Gainesville, Florida, United States of America; 3 Gulf Coast Research and Education Center, University of Florida, Wimauma, Florida, United States of America; 4 Department of Plant & Microbial Biology, University of California, Berkeley, California, United States of America; Kansas State University, United States of America

## Abstract

We investigated whether lines of transgenic tomato (*Solanum lycopersicum*) expressing the *Bs2* resistance gene from pepper, a close relative of tomato, demonstrate improved resistance to bacterial spot disease caused by *Xanthomonas* species in replicated multi-year field trials under commercial type growing conditions. We report that the presence of the *Bs2* gene in the highly susceptible VF 36 background reduced disease to extremely low levels, and VF 36-Bs2 plants displayed the lowest disease severity amongst all tomato varieties tested, including commercial and breeding lines with host resistance. Yields of marketable fruit from transgenic lines were typically 2.5 times that of the non-transformed parent line, but varied between 1.5 and 11.5 fold depending on weather conditions and disease pressure. Trials were conducted without application of any copper-based bactericides, presently in wide use despite negative impacts on the environment. This is the first demonstration of effective field resistance in a transgenic genotype based on a plant R gene and provides an opportunity for control of a devastating pathogen while eliminating ineffective copper pesticides.

## Introduction

Bacterial spot disease, a complex of four *Xanthomonas* species, is among the most widespread and destructive diseases of tomatoes and peppers throughout the world, causing lesions on aerial plant parts leading to defoliation and fruit loss [1]. It has chronically afflicted U.S. tomato production, particularly in Florida, where the largest production of fresh market tomatoes occurs. Ninety-seven per cent of Florida acres are affected, and yield losses may reach fifty per cent of marketable production [2].

Various crop protection compounds have been used to control bacterial spot in commercial tomato and pepper production. In the 1950s, streptomycin was commonly used to control plant diseases caused by bacteria, including bacterial spot. *Xanthomonas euvesicatoria* (Race T1), the prevalent bacterial spot race in Florida at the time, quickly became resistant to streptomycin [3], [4], and its use was discontinued. In the 1960s, fixed copper compounds and copper-fungicide mixes became the primary means of bacterial spot control. Initially fixed copper was used alone, but resistance in xanthomonads developed quickly [5]. In response to observing increased efficacy when copper was mixed with ethylenebisdithiocarbamate (EBDC) fungicides such as maneb and mancozeb, growers began mixing fixed copper products with EBDC fungicides for improved bacterial spot control [5]. However, even these copper-fungicide mixes have become ineffective against tomato races of the pathogen, especially under conditions of high humidity and warm temperatures that favor heavy disease pressure (eg [6], [7], [8], [9]; Table S1).

Because crop protection compounds do not control the copper tolerant *Xanthomonas* races responsible for this disease, genetic resistance against bacterial spot has been a priority in tomato breeding programs. These breeding efforts have been slowed by the complex genetics of resistance and changing races of the pathogen, and consequently there are no commercial varieties with effective resistance to *Xanthomonas*. One commonly bred form of plant disease resistance relies on the evolution of specific intracellular immune receptors encoded by disease resistance, or *R,* genes. *R* genes have been selected through conventional breeding for over 100 years [10]. They encode specific receptors that recognize gene products made by specific races of a given pathogen species. These pathogen components are termed effectors, and they contribute to pathogen virulence by suppressing or modulating host defenses in susceptible plant genotypes that lack a corresponding *R* gene [11].

AvrBs2 is an effector that is highly conserved in a number of *Xanthomonas* species that infect a wide range of plant hosts, including tomato [12]. Unlike other key effectors, it is present in all six races of the tomato bacterial spot disease complex (Table S2). Mutations in AvrBs2 can impair virulence, indicating that it plays an important role in pathogenicity and may be a good target for durable resistance [12].

AvrBs2 is recognized by the R protein Bs2, identified in pepper, a fellow member of the Solanaceae and close relative of tomato. In the current study, we tested whether *Bs2* transgenic tomato lines were effective against current field races of bacterial spot in multi-year field trials conducted in two commercial growing regions of Florida. Our results demonstrated that tomatoes carrying the *Bs2* gene and which received no bactericidal crop protection compounds had the highest disease resistance of all the genotypes tested and had significantly increased yields relative to controls.

## Results

### All Field Strains of Bacterial Spot are Recognized by Plants Carrying the Resistance Gene *Bs2*

We assessed *Xanthomonas* populations on tomato plants across the state of Florida, and partly into Georgia, to examine the race structure and prevalence of key effectors. We tested 377 samples collected across all five commercial tomato production regions and made race determinations based on the elicitation of a hypersensitive reaction (HR) on plant genotypes carrying R genes that specifically recognize key effectors present in certain *Xanthomonas* races (Table S3). Two of the six known tomato bacterial spot races were found (Table 1). These were the highly related *X. perforans* strains that are distinguished into separate races based on the presence (race T3) or absence (race T4) of the effector AvrXv3. AvrXv3 triggers an HR on T3-resistant tomato lines carrying the Xv3 locus, thus race T4 overcomes resistance on these lines. 115 of the 377 strains (30%) were found to be race T3, and the remaining 70% (262 strains) were race T4. All strains elicited an HR on the *Bs2* genotype, indicating the presence of AvrBs2.

**Table 1:** Survey of bacterial spot strains isolated from tomato plants throughout Florida production zones.

				Hypersensitive reaction (HR) on^a^:						Race determinations^b^		Bactericide Resistance^c^	
County	Production Zone	Fruit type	# Strains isolated	Bonny Best (Susc)	HI7998 (Rxv)	FL216 (Xv3)	3X-2-4 (Xv4)	VF36-Bs2(Bs2)	ECW pepper	T3	T4	St	Cu
Miami-Dade	I	Large fruit	15	0	0	0	15	15	15	0	15	0	15
Miami-Dade	I	Large fruit	20	0	0	0	20	20	20	0	20	19	20
Miami-Dade	I	Large fruit	13	0	0	0	13	13	13	0	13	0	13
Miami-Dade	I	Plum	20	0	0	19	20	20	20	19	1	0	20
Palm Beach	II	Heirloom varieties	14	0	0	0	14	14	14	0	14	0	14
Collier	III	Florida 47	20	0	0	6	20	20	20	6	14	0	20
Collier	III	Roma	20	0	0	8	20	20	20	8	12	0	20
Collier	III	Grape	20	0	0	0	20	20	20	0	20	0	20
Collier	III	Grape	18	0	0	0	18	18	18	0	18	0	18
Collier	III	Large fruit	19	0	0	1	19	19	19	11	8	0	19
Manatee	IV	Large fruit	20	0	0	2	20	20	20	2	18	0	20
Manatee	IV	Large fruit	20	0	0	5	20	20	20	5	15	0	20
Manatee	IV	Large fruit	20	0	0	15	20	20	20	15	5	0	20
Manatee	IV	Large fruit	20	0	0	5	20	20	20	5	15	1	20
Manatee	IV	Large fruit	20	0	0	4	20	20	20	4	16	0	20
Gadsden	V	Large fruit	20	0	0	10	20	20	20	10	10	0	20
Gadsden	V	Large fruit	19	0	0	9	19	19	19	9	10	0	19
Decatur, GA	V	Large fruit	19	0	0	15	19	19	19	15	4	0	19
Decatur, GA	V	Large fruit	20	0	0	0	20	20	20	0	20	0	20
Decatur, GA	V	Large fruit	20	0	0	6	20	20	20	6	14	0	20
Totals			377	0	0	115	377	377	377	115	262	20	377

a Plant lines and their relevant genotypes (in parentheses) used for testing are listed in Table S3.

b Race determinations were based on HR elicited by effectors listed in Table S2.

c Bactericides tested were streptomcyn (St) and copper (Cu). Numbers denote resistant strains.

We also examined bactericidal sensitivity of the isolates and found 20 of 377 strains (5%) collected in this survey were resistant to streptomycin. 100% of strains were resistant to copper.

### *Bs2* Confers no Adverse Effects

The transgenic VF 36 lines containing *Bs2* from pepper were previously characterized and described [13]. The lines have a single transgene insertion, and sequencing of the transgenic locus confirmed the expected sequence of the gene construct as reported. Because detection of *Bs2* mRNA is difficult, confirmation of transgene expression has been based on bioassays (bacterial growth curves and hypersensitive reaction (HR)) in transiently and stably transformed lines. Besides the effects on disease resistance and yield reported in the current study, no other effects of the *Bs2* gene were observed on general growth, development, morphology or other horticultural characteristics of plants. Similar results were observed with other *Bs2* lines in the VF 36 background and also five other tomato varieties into which the gene has been introduced (unpublished data).

### *Bs2* Transgenic Resistance Confers the Highest Level of Disease Resistance among Bacterial Spot-resistant Tomato Genotypes

**Figure 1:**
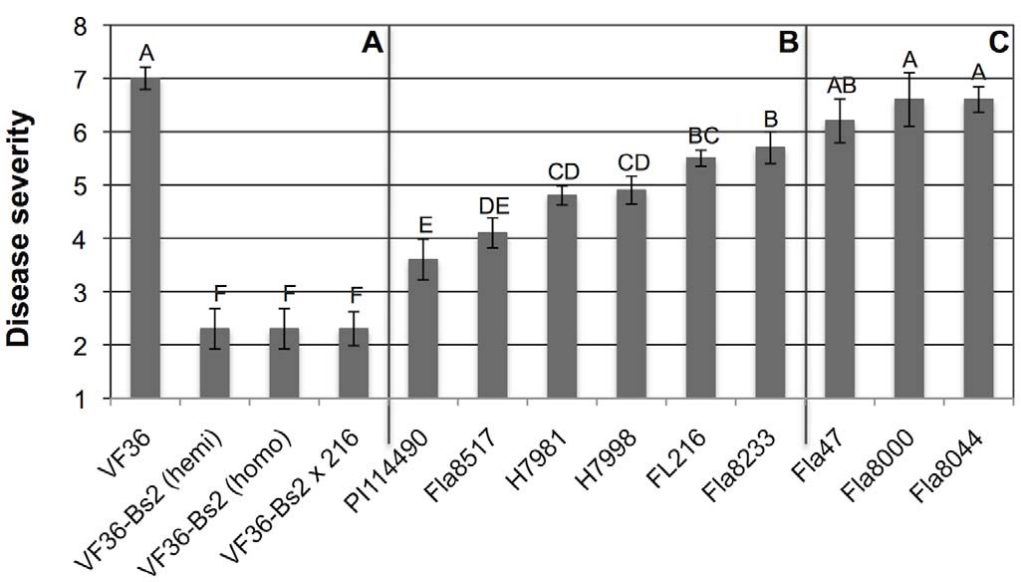
Comparison of bacterial spot disease severity among transgenic and disease resistant tomato genotypes. Results of field trials in Citra and Balm, FL, 2006-7. Data are overall mean disease severity scores from three field trials (Table S4). Panel a: VF 36 lines without *Bs2* or with one (hemi, and VF36-Bs2 x 216) or two (homo) copies of the *Bs2* gene; Panel b: tomato breeding lines with resistance to bacterial spot disease; Panel c: tomato lines susceptible to bacterial spot. Disease severity was determined by the Horsfall-Barratt defoliation scale (1 = 0%; 2 = 0–3%; 3 = 3–6%; 4 = 6–12%; 5 = 12–25%; 6 = 25–50%; 7 = 50–75%; 8 = 75–87%; 9 = 87–93%; 10 = 93–97%;11 = 97–100%; and 12 = 100% defoliation) [14]. Error bars denote standard errors of the mean. Letters above bars indicate statistically significant differences in mean values.

In a study comprised of three trials comparing thirteen tomato genotypes that included the best available resistant breeding lines, transgenic *Bs2* tomato lines consistently had the lowest disease symptoms in the presence of high *X. perforans* disease pressure (Fig. 1, Table S4). Whereas the non-transformed VF 36 line was the most susceptible with the highest disease severity score of 7, VF 36 lines carrying the *Bs2* gene had the lowest disease severity, with a rating of 2.3 (Fig. 1A). Transgenic lines that were hemizygous or homozygous for *Bs2* had the same disease severity scores, as did the F_1_ hybrid of VF 36-Bs2 crossed with FL216. Intermediate levels of disease severity were measured in several lines selected for conventional resistance to *Xanthomonas* with scores between 3.6 and 5.7 **(** Fig. 1B **)**. Notably the plant introduction accession PI 114490 showed the lowest disease severity of non-transgenic lines. This accession is used in several breeding programs, however successful transfer of its resistance loci into commercial varieties has not yet been accomplished. The lines Fla47, Fla. 8000, and Fla. 8044 were highly susceptible in our trials with scores between 6.2 and 6.7 **(** Fig. 1C **)**.

The measure of disease severity was the extent of bacterial spot symptoms on all plants in a plot, assessed using the Horsfall-Barratt scale [14]. Low levels of disease appeared as a small number of leaf spot lesions on a few leaves, with little defoliation. More extensive disease appeared as increasing numbers of lesions on a greater numbers of leaves, stems, and sometimes fruit. In the most severe cases, entire leaves or leaflets browned and dessicated from closely spaced lesions and associated necrosis.

### The *Bs2* Gene Confers a Substantial Yield Improvement under High Disease Pressure

**Figure 2:**
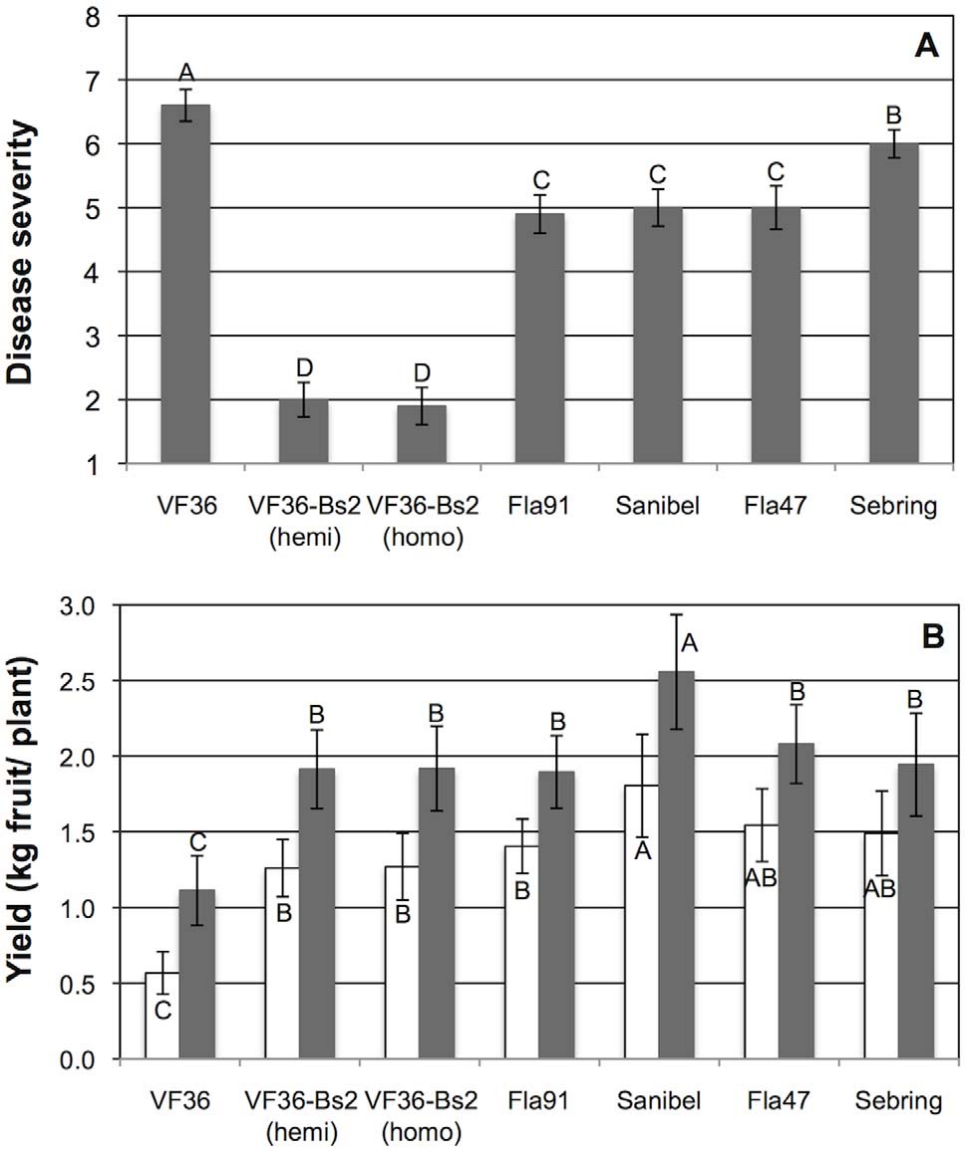
Comparison of disease severity and yield in transgenic and commercial tomato varieties. Results from Balm, FL, field trials, 2007–2010. Data shown are the combined analysis of five trials (Table S6), derived from the individual trial results given in Table S5. a: Bacterial spot disease severity. Disease severity was determined by the Horsfall-Barratt defoliation scale (See Fig. 1 legend) [14]. Error bars denote standard errors of the mean. Letters above bars indicate statistically significant differences in mean values. b: Yield. Marketable yield (open bars) is kg per plant for medium, large, and extra large fruit. Total yield (filled bars) is kg per plant for marketable yield plus small fruit and culls. VF 36 lines have no (VF36), one (VF36-Bs2 hemi) or two (VF36-Bs2 homo) copies of the *Bs2* gene. Error bars denote standard errors of the mean. Letters above bars indicate statistically significant differences in mean values for total yield data, whereas letters below bars indicate significant differences in mean values for marketable yield data.

In a second study to make performance comparisons between *Bs2* transgenic lines and standard commercial varieties, five trials were carried out to assess disease severity and yield impact **(** Fig. 2, Tables S5 (individual trial results) and S6 (combined trial results). As shown in Figure 2A, the lowest level of disease severity in these trials was again observed on transgenic lines, with scores of 2.0 and 1.9, for hemizygous and homozygous *Bs2* lines, respectively, contrasted with the high susceptibility of the non-transformed VF 36 line with a disease severity score of 6.6. All four of the commercial lines displayed appreciable disease symptoms with ratings between 4.9 and 6. In yield comparisons, the addition of *Bs2* into the VF 36 background substantially increased marketable and total yields of this un-adapted California variety to levels comparable to commercial Florida varieties **(** Fig. 2B **)**. VF 36 had the lowest marketable and total fruit yields of all the lines used in the trials, but the presence of *Bs2* in the VF 36 background boosted marketable yield from 0.06–1.49 kg/plant to 0.49–2.47 kg/plant, and total yields were increased from 0.12–2.40 kg/plant to 0.67–3.29 kg/plant.

Comparisons with non-inoculated, disease free plants were not possible under Florida field conditions where the disease is endemic.

### *Bs2* Confers a Positive Yield Effect Even under Low Disease Pressure

We considered the effect of temperatures and total rainfall on the five field experiments in Balm, FL **(** Table S7 **)**. Monthly temperatures were generally within 5% of seven-year averages, whereas rainfall was more variable, being either average, higher or lower than average, or seasonally average but unevenly distributed. Together with weather data, we considered the observed disease severity (pressure) and marketable yields **(** Table S5 **)** to determine an impact factor for *Bs2* in the VF 36 background (Table 2). *Bs2* typically conferred a yield enhancement of 2.5–2.8-fold and, in one season, a more than 10-fold increase. Even under very low disease pressure, VF 36-Bs2 lines displayed a 1.5-fold enhancement of marketable yield compared to VF 36 lines.

**Table 2:** Summary of field trial conditions at Balm, FL, and Bs2 impact factor.

	Fall 2007	Spring 2008	Fall 2008	Spring 2009	Fall 2010
**Temperature** a	Normal, hot late	Normal	Normal	Normal	Normal, cool late
**Rainfall** b	Average, uneven	Low-average	Low	Heavy wks 4–8	Very low
**Disease pressure** c	High	High	Low	High	Very low
**Yield** d	Low	Medium	Low-medium	Med-high	Very high
**Bs2 impact factor** e	11.8	2.8	2.5	2.6	1.5

a Normal: within 5% of typical averages (Table S7).

b Rainfall relative to typical monthly totals (Table S7).

c Relative disease pressure based on disease ratings in Table S5(A). High: VF 36 ratings above 7, commercial lines above 5; Low: VF 36 ratings below 6, commercial lines below 5.

d Relative yield summary based on marketable yields in Table S5(B). Low: 1–2 lbs/plant; Medium: 2.5–3.5 lbs/plant; High :4.5–6 lbs/plant. Fall 2008 estimates were approximated by doubling yield from single harvest.

e Bs2 impact was calculated by dividing the larger of the two values for marketable yield for VF36-Bs2 homo- or hemizygous plants with the marketable yield for VF 36 plants (Table S5(B)).

## Discussion

Bacterial spot disease has been a serious issue in tomato production for more than sixty years, and the commercial industry has been unable to control it via the extensive use of chemical, genetic, and cultural methods. We have investigated the potential of transgenic control of bacterial spot using the pepper *Bs2* gene in seven field trials under typical commercial growing conditions. The *Bs2* gene was identified more than two decades ago as an exceptional disease resistance candidate for transfer to tomato, because it activates resistance following specific recognition of AvrBs2, a *Xanthomonas* effector that is highly conserved in tomato races and races affecting other crops [12]. Despite periodic shifts in *Xanthomonas* races on field tomatoes and the expulsion or mutation of some effectors such as AvrXv3 under selection pressure, our survey showed that all current field isolates expressed active AvrBs2. Therefore, *Bs2* resistance targeted to AvrBs2 should be broadly effective.

The purpose of the current study was to examine the performance of selected *Bs2* lines under field conditions. Many publications on transgenic field trials report data at single locations, replicated, on average, over three seasons [15], [16], [17], [18], [19], [20], [21]. To provide a comprehensive view of *Bs2* field performance, we report seven trials in two locations on studies comparing (i) disease resistance relative to a set of genotypes with varying levels of resistance and (ii) disease resistance and yield relative to widely grown commercial cultivars. Trials were carried out in commercial growing zones IV (Balm) and V (Citra), where 32% and 6%, respectively, of Florida fresh market tomato production takes place.

We compared the disease severity of a range of tomato genotypes including standard commercial varieties, the best available spot resistant breeding lines, and *Bs2* transgenic lines. The non-transformed parent line VF 36 was the most susceptible to *X. perforans* infection including race T4, the predominant field race. The commercial cultivars Fla47, Fla91, Sebring and Sanibel were also highly susceptible. The plant introduction and inbred lines tested did express lower disease severity, however these lines have not been optimized for horticultural characteristics and require further breeding. VF 36 lines containing *Bs2* consistently exhibited the lowest bacterial spot disease symptoms of all genotypes and were generally free of characteristic lesions and defoliation.

In yield studies, *Bs2* had a significant positive impact on marketable and total yields. Whereas non-transformed VF 36 performed most poorly, with as little as one third of the production of Florida varieties, the presence of *Bs2* in the VF 36 background increased yields to levels comparable to commercial Florida varieties. These results are consistent with studies in pepper in which conventionally-bred bell and hot pepper varieties with the Bs2 locus were found to have lower disease severity and higher fruit yields in trials compared to non-Bs2 varieties [22], [23]. Both genetic background and weather conditions can effect disease severity and yield performance. However, despite varying environmental conditions across the trials, the presence of *Bs2* in the VF 36 background typically gave a 2.5-fold or greater increase in marketable yield.

The VF 36 tomato variety is highly susceptible to bacterial spot and not adapted to Florida growing conditions. It has been useful for proof of concept studies and for examining field performance of the trait, but it is not intended for commercial development. We have introduced the *Bs2* gene construct used in the current study, as well as others, into commercial tomato parent lines and hybrids from the University of Florida breeding program. Disease responses in the greenhouse and field fully replicate results with VF 36 lines, and preliminary field trials results demonstrate comparable yield increases in the Florida-adapted varieties (unpublished data). Yield increases of this magnitude are highly significant for tomato production in Florida.

**Figure 3:**
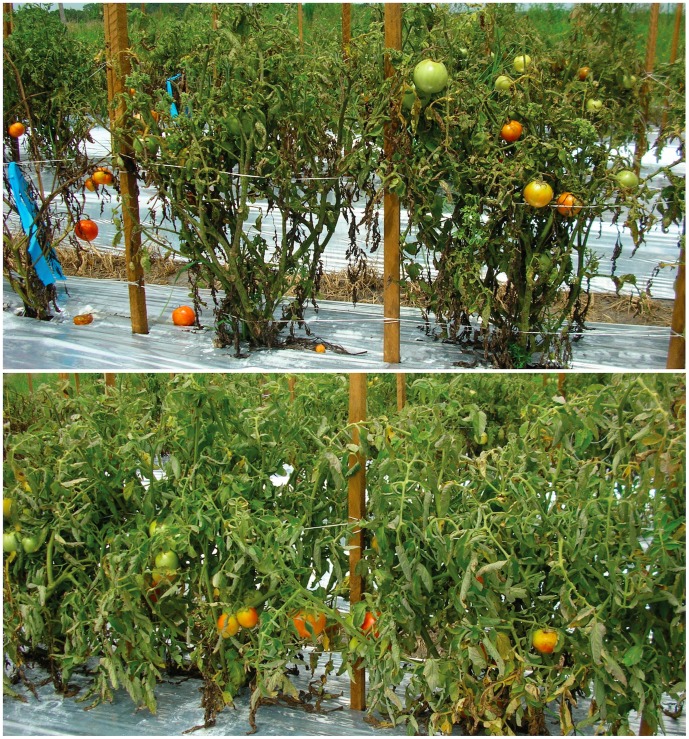
Photographs of non-transgenic and Bs2-transgenic VF36 lines in field trials. Top. Plants of the non-transformed VF36 line. Bottom. Plants of the transgenic VF36 line containing the 35S:Bs2 gene. Balm, FL, Spring 2008 Trial.

Yield differences may result from bacterial spot damage during early growth of tomato plants. In seasons that had hot, wet conditions conducive to infection during the first four to eight weeks that plants were in the field, the greatest spotting and defoliation were routinely observed. Later in the season, even heavily diseased plants tended to outgrow the disease, so that the lower half of the plants were often largely defoliated while the upper half had healthy foliage and fruit production **(** Fig. 3, top**)**. The net result was that fruit set on the bottom half of each plant was significantly impaired, reducing yield. In contrast, transgenic plants had full foliage and fruit set throughout the plant (Fig. 3, bottom). The current data do not allow us to distinguish if the observed yield increases are a direct effect of disease reduction by *Bs2* or if a separate mechanism is contributing to yield. That distinction would have to be made by evaluating yield differences caused by *Bs2* in the absence of bacterial spot. Due to the endemic nature of the disease in Florida, it is not possible to grow disease free plants in field trials, and so it could not be tested in the current study. Such a study would require a crop production greenhouse that excludes bacterial spot.

Our results show that transgenic disease resistance is an effective means of controlling bacterial spot. Given that all current field strains were found to be resistant to copper, it is clear why copper compounds have become ineffective for bacterial spot control, yet the lack of alternative measures has resulted in the continued broad use of copper pesticides. We note that streptomycin resistant strains were found in two of our survey collection areas, indicating renewed use of this bactericide in some fields. Given the rapid spread of resistance in the 1950s among *X. euvesicatoria* strains, it’s likely that any continued use of streptomycin will lead to resistance among current *X. perforans* strains as well.

Chemical control methods for bacterial spot are not simply ineffective but potentially hazardous as well. Frequent widespread application of copper compounds on tomatoes and citrus has caused persistent high levels of copper in Florida soils, which can leach into streams and ground water [24]. Levels are known to accumulate in wastewater from washing tomatoes at packing houses [25], exceeding safe drinking water limits established by the U. S. Environmental Protection Agency (http://water.epa.gov/drink/contaminants/basicinformation/copper.cfm). In fact fixed copper compounds are amongst the oldest and most toxic crop protection compounds used in both conventional and organic agriculture (www.nysipm.cornell.edu/publications/eiq/files/EIQ_values_2010.pdf). Certainly crop protection compounds play a crucial role in global food production, however safer alternatives must be sought for chemicals that are ineffective or hazardous. In light of the environmental and health issues associated with copper compounds and the fact that transgenic disease resistant papaya and squash have been commercially available and safely consumed for more than a decade [26], one must consider that transgenic disease resistance may provide an improved means of controlling bacterial spot.

Our study is the first demonstration of transgenic resistance in replicated multi-year field trials based on the use of a plant R gene. The Bs2 R protein is a member of the nucleotide binding site-leucine-rich repeat (NB-LRR) family of innate immune receptors [13], one of the largest families of plant proteins [27], [28]. Because *Bs2* occurs in pepper, the Bs2 protein, like many NB-LRR proteins, has been widely consumed with no known toxicity or allergenicity. The risk of transgene escape is low, because tomato is >99% self-pollinating [29] and no sexually compatible relatives occur in North America. These features suggest a good safety profile for *Bs2* transgenic tomatoes, but before a commercial product can be released all appropriate regulatory studies and requirements will be fulfilled.

Transgenic *Bs2* tomatoes provide a feasible alternative for improving both yields and tomato production practices in areas of chronic bacterial spot infection. Proper stewardship of this useful trait should include combining it with other resistance genes for bacterial spot. Even though AvrBs2 is an important contributor to pathogenicity, deployment of *Bs2* will put pressure on *Xanthomonas* strains to evolve this effector to overcome resistance. Indeed strains have been isolated with *AvrBs2* mutations that recover the ability to grow on pepper and tomato plants carrying the *Bs2* gene [30], [31], [32]. However these strains show impaired virulence on both hosts in the presence and absence of *Bs2,* retain wild-type copies of the gene in populations, and may be unable to compete in the field with wild-type strains. Regardless of the potential for durability of *Bs2,* or indeed any disease resistance gene, we strongly advocate deploying it in a genetic background with good general disease resistance and with additional specific resistance genes, preferably stacked at a single locus. Finally, use of cultural practices to minimize spread of the pathogen will continue to be important in limiting the impact of bacterial spot.

## Materials and Methods

### Plant Lines

Plant lines used in this study are listed in Table S3. Transgenic tomato plants were as described [13] using the VF 36 variety transformed with the pepper Bs2 cDNA sequence driven by the CaMV 35S promoter. Three transgenic lines were used, a homozygous VF 36-Bs2 line, a hemizygous VF 36-Bs2 line, prepared by backcrossing the homozygous line to VF 36, and an additional cross of the VF 36-Bs2 line with Florida 216 (FL216), producing VF 36-Bs2x216. Commercial tomato varieties used in trials were Florida 47 (Fla47), Florida 91 (Fla91), Sebring and Sanibel. Experimental inbred lines, Hawaii 7998 and 7981 (H7998, H7981) and plant introduction accessions, PI 114490 and PI 128216, or their derivatives (FL216, produced by crossing PI 128216 with Fla7060) with reported resistance to *Xanthomonas* were also used in trials. The University of Florida breeding program supplied four inbred lines (Fla. 8000, Fla. 8044, Fla. 8233, Fla. 8517) that were included in trials. For the survey of bacterial strains, the tomato lines Bonny Best, H7998, FL216, 3x-2-4, and VF 36-Bs2, and the pepper variety Early Calwonder (ECW) were used to identify bacterial races. The tomato variety Bonny Best is susceptible to *Xanthomonas*, whereas tomato lines H7998, FL216, 3x-2-4, and VF 36-Bs2 contain resistance loci Rxv, Xv3, Xv4, and Bs2, respectively [13], [33], [34], [35], [36], [37]. These resistance loci trigger a hypersensitive reaction (HR) in response to strains containing the corresponding effectors AvrRxv, AvrXv3, AvrXv4, and AvrBs2, respectively. Differential HR results were used to determine which effectors were present in each strain (Table S2) and permit the identification of the race. The pepper variety ECW can distinguish between strains that can grow on pepper (T1, T2 and *X. gardneri*) or cannot (T3-5).

### Field Survey of Florida *Xanthomonas* Strains

Bacterial samples were isolated from bacterial spot lesions from twenty different leaflets per field, randomly collected from twenty unique field locations throughout the five major production zones in Florida between October and December, 2006. Production zone V extends partially into Georgia. Three hundred seventy-seven individual isolates were grown on nutrient agar for 24 hr at 28°C, and bacterial cells were removed and suspended in sterile tap water. Suspensions were adjusted to a concentration of 10^8^ cfu/ml and inoculated by infiltration using a hypodermic needle and syringe on to a panel of tomato and pepper plants of varying resistance genotypes (Tables 1 and S3). Each plant was scored as producing a hypersensitive reaction (HR) after 1–2 days, or as producing disease at 3 days.

The bacterial isolates were also tested for sensitivity to the bactericides, streptomycin and copper using established procedures. All strains were tested for growth on media with either streptomycin sulfate (200 µg/ml) or copper sulfate (pentahydrate) 200 µg/ml (0.8 mM)) after 3 days [3], [38].

### Field Trials, Inoculation and Assessment

Tomato plants were seeded in a greenhouse and grown for 4–6 weeks prior to transplanting to the field. Young plants were either inoculated with *X. perforans* strains in the greenhouse and then transferred to the field after one week (2006, 2007), or first transplanted and then inoculated (2008–2010). Inoculations were conducted with either race T3 (2006) or T4 (in 2007–2010) and were achieved by spraying the plants with a bacterial suspension adjusted to 10^6^ cfu/ml in 0.025% (v/v) of Silwet L77. After completing the bacterial survey in 2006 and learning that the T4 race predominated in field trials, and because it is highly related to race T3 except it has the added ability to grow on race 3 resistant tomato hosts, we used only T4 as inoculum in all subsequent field trials. Trials occurred at the University of Florida Science Research Unit in Citra, FL (2006, 2007) and Gulf Coast Research and Education Center in Balm, FL (2007–2010). Trials were randomized complete block designs with 3–4 blocks of up to nineteen genotypes per block depending on the trial. Field plots were prepared using standard tillage practices and maintained during the trial period with standard fertilizer and pesticide regimes [39] except no bactericidal compounds were applied. Disease ratings were made at various intervals throughout the growing period. Assessments were made by estimating percent disease symptoms and defoliation caused by bacterial spot using the Horsfall-Barratt scale, where 1 = 0%; 2 = 0–3%; 3 = 3–6%; 4 = 6–12%; 5 = 12–25%; 6 = 25–50%; 7 = 50–75%; 8 = 75–87%; 9 = 87–93%; 10 = 93–97%;11 = 97–100%; and 12 = 100% defoliation [14]. Yield determinations were made by harvesting mature-green and riper fruit from each plot. Fruit were counted and weighed for culls, small, medium, large and extra large sizes with the latter three categories comprising marketable yield, and all categories combined for total yield. Generally two harvests were made, with the exception of the Fall 2008 trial where only one harvest was made and with the exception of the Fall 2007 trial in which no fruit was harvested from the cultivar Sanibel. All field trials were carried out under Notification from the US Department of Agriculture and in accordance with an approved design protocol.

The seven trials were carried out in two locations in the Spring or Fall planting season. In one series of trials, consisting of plots at Citra (Fall 2006, Fall 2007) and Balm (Fall 2007), the extent of disease in transgenic lines was compared with ten other tomato genotypes having a range of resistance to bacterial spot. In the other series, data from five trials at the Balm Experiment Station (Fall 2007 (which had a subset of plants in common with the disease resistance series mentioned above), Spring 2008, Fall 2008, Spring 2009, and Fall 2010) were collected to examine the correlation between the extent of disease incidence and impact on yield in transgenic lines and commonly planted commercial cultivars.

### Statistical Analysis

Disease ratings and yield data were evaluated with analysis of variance using PROC GLM (SAS Institute 1999) and means separated with a Duncan multiple range test (P<0.05).

### Seasonal Conditions and Bs2 Impact Factor

Weather data was retrieved using the Florida Automated Weather Network (FAWN) website (fawn.ifas.ufl.edu*)*. Typical monthly temperatures and rainfall were determined by averaging data for each month over a seven year period from 2004, the earliest year for which archived data was available, through 2010. For each month of a given year in which plants were in the field, monthly temperature and rainfall data were recorded and also expressed as a percentage of the seven-year monthly average.

## Supporting Information

Table S1 **Field trial reports of copper effectiveness on bacterial spot disease on tomato.**
(DOCX)

Table S2 **Bacterial spot disease races of tomato and occurrence of key effectors for resistance breeding.**
(DOCX)

Table S3 **Tomato and pepper lines used in field trials and race determinations.**
(DOCX)

Table S4 **Comparison of bacterial spot disease severity in tomato lines in Florida field trials.**
(DOCX)

Table S5 **Comparison of disease severity and yield in transgenic and commercial tomato lines in Balm, Florida, field trials.**
(DOCX)

Table S6 **Combined field trial analysis for Balm, FL.**
(DOCX)

Table S7 **Temperature and rainfall data for trials in Balm, FL.**
(DOCX)
