# Supplementary material for: Transgenic Resistance Confers Effective Field Level Control of Bacterial Spot Disease in Tomato
Source: PLoS One. 2012 Aug 1;7(8):e42036. doi: 10.1371/journal.pone.0042036 (PMC3411616; doi:10.1371/journal.pone.0042036)
Supplement: Table S1 — Field trial reports of copper effectiveness on bacterial spot disease on tomato. (DOCX) [file pone.0042036.s001.docx]

**Table S1: Field trial reports of copper effectiveness on bacterial spot disease on tomato.**

|  | |  |  | **Significant difference between treatment and untreated control** | | |  |  |
| --- | --- | --- | --- | --- | --- | --- | --- | --- |
| **Year** | **Location** | | **Copper treatment** | **Foliar Rating^a^** | **AUDPC^b^** | **Marketable Yield^c^** | **Ref** | |
| 2007 | | OH | Copper hydroxide + maneb^d^ | No | No | No | [[1](#_ENREF_1)] |  |
| 2008 | | OH | Copper hydroxide + maneb | No | No | No | [[2](#_ENREF_2)] |  |
| 2008 | | TN | Copper hydroxide | No | ND^e^ | No | [[3](#_ENREF_3)] |  |
| 2009 | | OH | Copper hydroxide | Yes^f^ | No | No | [[4](#_ENREF_4)] |  |
| 2009 | | VA | Copper hydroxide + Serenade Max^g^ | No | ND | No | [[5](#_ENREF_5)] |  |
|  | |  | Copper hydroxide + QRD146^g^ | No | ND | No |  |  |
| 2010 | | FL | Copper sulfate + mancozeb^d^ | No | No | No | [[6](#_ENREF_6)] |  |
|  | |  | Copper hydroxide | No^h^ | Yes^h^ | No |  |  |
| 2010 | | FL | Copper hydroxide + mancozeb | No | No | ND | [[7](#_ENREF_7)] |  |
| 2010 | | FL | Copper sulfate + mancozeb | No | No | No | [[8](#_ENREF_8)] |  |
| 2010 | | FL | Copper sulfate + mancozeb | No | No | No | [[9](#_ENREF_9)] |  |

^a^ Foliar ratings were reported as Horsfall-Barratt ratings or percent diseased foliage.

^b^ Area under the disease progress curve.

^c^ Total of medium, large, and extra-large fruit.

^d^ Maneb and mancozeb are ethylenebisdithiocarbamate (EBDC) fungicides.

^e^ ND, Not determined.

^f^ Foliar symptoms were lower on copper treated plots. Fruit symptoms were not.

^g^  Serenade Max and QRD146 are biopesticides comprised of non-pathogenic bacterial strains, sold by AgraQuest, Davis, CA.

^h^ Early season ratings showed decreased disease symptoms but later ratings were not different. Disease development, assessed by AUDPC, was significantly lower with copper treatment.

1. Miller SA, Mera JR, Xu X, Baysal F (2008) Evaluation of fungicides and bactericides for the control of foliar and fruit diseases of processing tomatoes, 2007. Plant Disease Management Reports 2: V037.

2. Miller SA, Mera JR (2009) Evaluation of fungicides and bactericides for the control of foliar and fruit diseases of processing tomatoes, 2008. Plant Disease Management Reports 3: V008.

3. Canaday CH (2009) Effects of cultivar, a SAR inducer, and fungicide reduction on disease and yield of staked-tomatoes, 2008. Plant Disease Management Reports 3: V053.

4. Miller SA, Mera JR, Baysal-Gurel F (2010) Evaluation of fungicides and bactericides for the control of foliar and fruit diseases of processing tomatoes, 2009. Plant Disease Management Reports 4: V011.

5. Rideout SL, Waldenmaier CM, Wimer AF, Stapleton JB (2010) Evaluation of selected fungicides for the management of bacterial spot in tomato, 2009. Plant Disease Management Reports 4: V028.

6. Vallad GE, Huang CH (2011) Evaluation of bacteriocides and Actigard for management of bacterial spot of tomato, Spring 2010. Plant Disease Management Reports 5: V067.

7. Zhang S, Mersha Z, Fu Y (2011) Field evaluation of Actigard for bacterial spot disease management on tomato in South Florida, 2010. Plant Disease Management Reports 5: V016.

8. Vallad GE, Huang CH (2011) Evaluation of Quintec for management of bacterial spot of tomato, Spring 2010. Plant Disease Management Reports 5: V061.

9. Huang CH, Vallad GE (2011) Evaluation of Actigard for management of bacterial spot of tomato, Spring 2010. Plant Disease Management Reports 5: V066.
