# Supplementary material for: Transgenic Resistance Confers Effective Field Level Control of Bacterial Spot Disease in Tomato
Source: PLoS One. 2012 Aug 1;7(8):e42036. doi: 10.1371/journal.pone.0042036 (PMC3411616; doi:10.1371/journal.pone.0042036)
Supplement: Table S2 — Bacterial spot disease races of tomato and occurrence of key effectors for resistance breeding. (DOCX) [file pone.0042036.s002.docx]

**Table S2: Bacterial spot disease races of tomato and occurrence of key effectors for resistance breeding.**

|  | **Effector occurrence** ^a,^ [[1](#_ENREF_1)] | | | |  |
| --- | --- | --- | --- | --- | --- |
| **Race** | **AvrRxv** | **AvrXv3** | **AvrXv4** | **AvrBs2** | **Xanthomonas species** |
| T1^b^ | + | - | - | + | *euvesicatoria* (Xcv) |
| T2 | - | - | - | + | *vesicatoria* (Xv) |
| T3 | - | + | + | + | *perforans* (Xp) |
| T4 | - | - | + | + | *perforans* (Xp) |
| T5 | - | - | - | + | *perforans* (Xp) |
| ^c^ | - | - | - | + | *gardneri*  (Xg) |

^a^ + present or – absent in each race

^b^  T indicates tomato races

^c^ No T race designation

1. Potnis N, Krasileva K, Chow V, Almeida NF, Patil PB, et al. (2011) Comparative genomics reveals diversity among xanthomonads infecting tomato and pepper. BMC Genomics 12: 146.
