# Supplementary material for: Transgenic Resistance Confers Effective Field Level Control of Bacterial Spot Disease in Tomato
Source: PLoS One. 2012 Aug 1;7(8):e42036. doi: 10.1371/journal.pone.0042036 (PMC3411616; doi:10.1371/journal.pone.0042036)
Supplement: Table S3 — Tomato and pepper lines used in field trials and race determinations. (DOCX) [file pone.0042036.s003.docx]

**Table S3. Tomato and pepper lines used in field trials and race determinations.**

| **Variety** | **Type** | **Source** | **Bacterial Spot Race Resistance** | **Bacterial Spot Resistance Loci** | **Reference** |
| --- | --- | --- | --- | --- | --- |
| VF 36 | CA large round FM | Scott | None | None |  |
| VF 36-Bs2 (homozygote) | CA large round FM | Jones/ Stall | T1-5, *X. gardneri* | Bs2 | [[1](#_ENREF_1)] |
| VF 36-Bs2 (hemizygote) | CA large round FM | Jones/ Stall | T1-5, *X. gardneri* | Bs2 | [[1](#_ENREF_1)] |
| Fla47 | FL Large round FM hybrid | Seminis | None | None |  |
| Fla91 | FL Large round FM hybrid | Seminis | None | None |  |
| Sebring | FL Large round FM hybrid | Syngenta | None | None |  |
| Sanibel | FL Large round FM hybrid | Seminis | None | None |  |
| H7998 | Hawaiian inbred | Stall | T1 | Rxv1-3 | [[2](#_ENREF_2),[3](#_ENREF_3),[4](#_ENREF_4)] |
| H7981 | Hawaiian inbred | Stall | T3 | Xv3 | [[5](#_ENREF_5),[6](#_ENREF_6)] |
| PI 114490 | Indeterminate yellow cherry PI accession | D. Francis  Ohio State | T1-4 | QTL | [[7](#_ENREF_7),[8](#_ENREF_8)] |
| FL216 | *L.* *pimpinellifolium* accession PI128216 crossed with FL7060 | Stall | T3 | Xv3 | [[9](#_ENREF_9)] |
| VF36-Bs2x216 | Cross of VF 36-Bs2 (homozygote) and FL216 | Jones/ Stall | T1-5, *X. gardneri* | Bs2, Xv3 |  |
| Fla. 8000 | FL Large round FM parent line | Scott | T3 | Xv3 | [[10](#_ENREF_10)] |
| Fla. 8044 | FL Large round FM parent line | Scott | None | None |  |
| Fla. 8233 | FL Large round FM parent line, derived from PI114490 and HI7998 | Scott | T3-4 | Xv3 | [[11](#_ENREF_11)] |
| Fla. 8517 | FL Roma breeding line | Scott | T3-4 | Xv3 | [[11](#_ENREF_11)] |
| 3X-2-4 | FL large round FM parent line | Stall | T3-4 | Xv4 | [[12](#_ENREF_12)] |
| Bonny Best | Indeterminate medium round | Stall | None | None |  |
| ECW | Bell pepper | Stall | T1, T2, *X. gardneri* | unknown | [[13](#_ENREF_13)] |

Abbreviations: CA, California; FM, fresh market; FL, Florida; PI, plant introduction, QTL, quantitative trait locus conferring quantitative resistance; Rxv1-3, Xv3, Xv4, Bs2, resistance genes detecting the effectors AvrRxv, AvrXv3, AvrXv4, and AvrBs2, respectively. See Table S2 for description of bacterial spot races and effector composition.

1. Tai TH, Dahlbeck D, Clark ET, Gajiwala P, Pasion R, et al. (1999) Expression of the Bs2 pepper gene confers resistance to bacterial spot disease in tomato. Proc Natl Acad Sci USA 96: 14153-14158.

2. Scott JW, Jones JB, Somodi GC (2001) Inheritance of resistance in tomato to race T3 of the bacterial spot pathogen. J Amer Soc Hort Sci 126: 436-441.

3. Scott JW, Stall RE, Jones JB, Somodi GC (1996) A single gene controls the hypersensitive response of Hawaii 7981 to race 3 (T3) of the bacterial spot pathogen. Rpt Tomato Genet Coop 46.

4. Wang J-F, Jones JB, Scott JW, Stall RE (1994) Several genes in Lycopersicon esculentum control hypersensitivity to Xanthomonas campestris pv. vesicatoria. Phytopathology 84: 702-706.

5. Jones JB, Scott JW (1986) Hypersensitive response in tomato to Xanthomonas campestris pv. vesicatoria. Plant Disease 70: 337-339.

6. Scott JW, Jones JB (1989) Inheritance of resistance to foliar bacterial spot of tomato incited by Xanthomonas campestris pv. vesicatoria. J Amer Soc Hort Sci 114: 111-114.

7. Scott JW, Francis DM, Miller SA, Somodi GC, Jones JB (2003) Tomato bacterial spot resistance derived from PI114490; Inheritance of resistance to Race T2 and relationship across three pathogen races. J Amer Soc Hort Sci 128: 698-703.

8. Scott JW, Hutton SF, Jones JB, Francis DM, Miller SA (2006) Resistance to bacterials spot race T4 and breeding for durable and broad resistance to other races. Rpt Tomato Genet Coop 56: 33-36.

9. Robbins MD, Darrigues A, Sim S-C, Masud MAT, Francis DM (2009) Characterization of hypersensitive resistance to bacterial spot Race T3 ( Xanthomonas perforans) from tomato accession PI 128216. Phytopathology 99: 1037-1044.

10. Yang WC, Francis DM (2007) Genetics and breeding for resistance to bacterial diseases in tomato. In: Razdan MK, Mattoo AK, eds. Genetic Improvement of Solanaceous Crops. Enfield, NH, USA: Science Publishers. pp. 379-419.

11. Hutton SF, Scott JW (2010) Inheritance of resistance to bacterial spot Race T4 from three tomato breeding lines with differing resistancebackgrounds. J Amer Soc Hort Sci 135: 150-158.

12. Astua-Monge G, Minsavage GV, Stall RE, Vallejos CE, Davis MJ, et al. (2000) Xv4-vrxv4: a new gene-for-gene interaction identified between Xanthomonas campestris pv. vesicatoria race T3 and wild tomato relative Lycopersicon pennellii. Molecular Plant-Microbe Interactions 13: 1346-1355.

13. Minsavage G, Dahlbeck D, Whalen M, Kearney B, Bonas U, et al. (1990) Gene-for-gene relationships specifying disease resistance in Xanthomonas campestris pv. vesicatoria -pepper interactions. Mol Plant Microb Interact 3: 41-47.
