# Supplementary material for: Transgenic Resistance Confers Effective Field Level Control of Bacterial Spot Disease in Tomato
Source: PLoS One. 2012 Aug 1;7(8):e42036. doi: 10.1371/journal.pone.0042036 (PMC3411616; doi:10.1371/journal.pone.0042036)
Supplement: Table S4 — Comparison of bacterial spot disease severity in tomato lines in Florida field trials. (DOCX) [file pone.0042036.s004.docx]

**Table S4: Comparison of bacterial spot disease severity in tomato lines in Florida field trials.**

|  | **Fall 2006 Citra** | **Fall 2007 Citra** | **Fall 2007 Balm^1^** | **Overall** |
| --- | --- | --- | --- | --- |
| **Plant Line** | **Mean^2^** | **Mean** | **Mean** | **Mean** |
| VF 36-Bs2 x 216 | 1.2^E^ | 2.0^D^ | 3.6^EF^ | 2.3^F^ |
| VF 36-Bs2 (homo) | 1.0^E^ | 2.0^D^ | 4.0^E^ | 2.3^F^ |
| VF 36-Bs2 (hemi) | 1.0^E^ | 2.0^D^ | 4.0^E^ | 2.3^F^ |
| PI 114490 | 2.7^D^ | 4.7^C^ | 3.4^F^ | 3.6^E^ |
| Fla. 8517 | 4.0^CD^ | 4.5^C^ | 3.9^EF^ | 4.1^DE^ |
| HI7981 | 4.5^BC^ | 4.5^C^ | 5.4^D^ | 4.8^CD^ |
| HI7998 | 4.5^BC^ | 4.5^C^ | 5.6^CD^ | 4.9^CD^ |
| FL216 | 5.2^A-C^ | 5.2^C^ | 6.0^BC^ | 5.5^BC^ |
| Fla. 8233 | 5.0^A-C^ | 6.5^B^ | 5.6^CD^ | 5.7^B^ |
| Fla47 | 5.2^A-C^ | 8.0^A^ | 5.5^CD^ | 6.2^AB^ |
| Fla. 8000 | 4.0^CD^ | 8.0^A^ | 5.7^B-D^ | 6.6^A^ |
| Fla. 8044 | 6.0^AB^ | 7.5^A^ | 6.2^B^ | 6.6^A^ |
| VF 36 | 6.5^A^ | 7.5^A^ | 7.0^A^ | 7.0^A^ |

^1^ *Alternaria* present in this trial produced bacterial spot-like disease symptoms and higher than typical scores, including on Bs2 lines.

**^2^** Disease severity scores were determined using the Horsfall-Barratt scale (Fig. 1). Treatment differences were determined using the Waller-Duncan T-test (p<0.05). Mean values with identical letters were not significantly different.
