# Supplementary material for: Transgenic Resistance Confers Effective Field Level Control of Bacterial Spot Disease in Tomato
Source: PLoS One. 2012 Aug 1;7(8):e42036. doi: 10.1371/journal.pone.0042036 (PMC3411616; doi:10.1371/journal.pone.0042036)
Supplement: Table S5 — Comparison of disease severity and yield in transgenic and commercial tomato lines in Balm, Florida, field trials. (DOCX) [file pone.0042036.s005.docx]

**Table S5: Comparison of disease severity and yield in transgenic and commercial tomato lines in Balm, Florida, field trials.**

**A. Bacterial spot disease severity**

|  | **Fall 2007** | **Spring 2008** | **Fall 2008** | **Spring 2009** | **Fall 2010** |
| --- | --- | --- | --- | --- | --- |
|  | **Mean^1, 2^** | **Mean** | **Mean** | **Mean** | **Mean** |
| **Plant Line** |  |  |  |  |  |
| VF 36-Bs2 (homo) | 4.0^D^ | 1.7^C^ | 1.5^C^ | 1.0^C^ | 1.0^D^ |
| VF 36-Bs2 (hemi) | 4.0^D^ | 1.7^C^ | 2.0^C^ | 1.0^C^ | 1.0^D^ |
| Fla91 | 5.5^C^ | 5.7^B^ | 4.0^B^ | 5.7^B^ | 3.3^C^ |
| Sanibel | 6.0^BC^ | 5.7^B^ | 3.7^B^ | 5.7^B^ | 3.3^C^ |
| Fla47 | 5.5^C^ | 6.5^AB^ | 4.0^B^ | 5.3^B^ | 3.3^C^ |
| Sebring | 6.2^B^ | 6.7^A^ | 6.0^A^ | 6.0^B^ | 4.5^B^ |
| VF 36 | 7.0^A^ | 7.2 ^A^ | 5.2^AB^ | 7.7^A^ | 5.8^A^ |

**B. Yield**

|  | **Fall 2007** | | **Spring 2008** | | **Fall 2008** | | **Spring 2009** | | **Fall 2010** | |
| --- | --- | --- | --- | --- | --- | --- | --- | --- | --- | --- |
|  | **Marketable Yield^2, 3^** | **Total Yield** | **Marketable Yield** | **Total Yield** | **Marketable Yield**  ^3^ | **Total Yield** ^3^ | **Marketable Yield** | **Total Yield** | **Marketable Yield** | **Total Yield** |
| **Plant Line** |  |  |  |  |  |  |  |  |  |  |
| VF 36-Bs2 (homo) | 0.53^BC^ | 0.90^B^ | 1.12^A^ | 2.27^A^ | 0.50^A^ | 0.68^AB^ | 2.47^A^ | 3.29^A^ | 2.28^B^ | 3.09^B-D^ |
| VF 36-Bs2 (hemi) | 0.75 ^AB^ | 1.11^AB^ | 1.25^A^ | 2.67^A^ | 0.49^A^ | 0.67^AB^ | 1.99^A^ | 2.70^A-C^ | 2.24^B^ | 2.84^CD^ |
| Fla91 | 0.88^AB^ | 1.02^AB^ | 1.58^A^ | 2.31^A^ | 0.43^AB^ | 0.64^A-C^ | 2.13^A^ | 3.03^AB^ | 2.11^B^ | 2.61^CD^ |
| Sanibel | ND^4^ | ND^4^ | 1.37^A^ | 2.39^A^ | 0.48^A^ | 0.86^A^ | 1.85^A^ | 2.58^A-C^ | 3.65^A^ | 4.46^A^ |
| Fla47 | 1.23^A^ | 1.50^A^ | 1.13^A^ | 2.28^A^ | 0.44^AB^ | 0.64^A-C^ | 1.94^A^ | 2.43^A-C^ | 3.21^A^ | 3.67^A-C^ |
| Sebring | 0.71^AB^ | 0.87^B^ | 1.53^A^ | 2.30^A^ | 0.28^AB^ | 0.43^BC^ | 1.79^A^ | 2.30^BC^ | 3.38^A^ | 4.05^AB^ |
| VF 36 | 0.06^C^ | 0.12^C^ | 0.45^B^ | 1.45^B^ | 0.20^B^ | 0.31^C^ | 0.95^B^ | 1.77^C^ | 1.49^B^ | 2.40^D^ |

**^1^** Disease severity scores were determined using the Horsfall-Barratt scale (Fig. 1). *Alternaria* present in the Fall 2007 trial produced bacterial spot-like disease symptoms and higher than typical scores, including on Bs2 lines.

^2^ Treatment differences were determined using the Waller-Duncan T-test (p<0.05). Mean values with identical letters were not significantly different.

^3^ Yields are kg per plant. Marketable yield is medium, large, and extra large fruit. Total yield is all fruit including small fruit and culls. Except for the Fall 2008 trial, all trials were harvested two times and yield measurements are the total of both harvests. The Fall 2008 trial was harvested only once.

^4^ Not determined.
