# Supplementary material for: Transgenic Resistance Confers Effective Field Level Control of Bacterial Spot Disease in Tomato
Source: PLoS One. 2012 Aug 1;7(8):e42036. doi: 10.1371/journal.pone.0042036 (PMC3411616; doi:10.1371/journal.pone.0042036)
Supplement: Table S6 — Combined field trial analysis for Balm, FL. (DOCX) [file pone.0042036.s006.docx]

**Table S6: Combined field trial analysis for Balm, FL.**

|  | **2007-2010**^1^ | | |
| --- | --- | --- | --- |
|  | **Disease rating^2,3^** | **Marketable Yield (kg)^3,4^** | **Total Yield (kg)^3,4^** |
| **Plant Line** |  |  |  |
| VF 36-Bs2 (homo) | 1.9^D^ | 1.27^B^ | 1.92^B^ |
| VF 36-Bs2 (hemi) | 2.0^D^ | 1.26^B^ | 1.91^B^ |
| Fla91 | 4.9^C^ | 1.40^B^ | 1.89^B^ |
| Sanibel | 5.0^C^ | 1.80^A^ | 2.56^A^ |
| Fla47 | 5.0^C^ | 1.54^AB^ | 2.08^B^ |
| Sebring | 6.0^B^ | 1.49^AB^ | 1.94^B^ |
| VF 36 | 6.6^A^ | 0.57^C^ | 1.11^C^ |

^1^ Combined analysis of 2007-2010 data presented in Table S5.

^2^ Disease severity scores were determined using the Horsfall-Barratt scale (Fig. 1).

^3^ Treatment differences were determined using the Waller-Duncan T-test (p<0.05). Mean values with identical letters were not significantly different.

^4^ Yields are kg per plant. Marketable yield is medium, large, and extra large fruit. Total yield is all fruit including small fruit and culls.
