# Supplementary material for: Transgenic Resistance Confers Effective Field Level Control of Bacterial Spot Disease in Tomato
Source: PLoS One. 2012 Aug 1;7(8):e42036. doi: 10.1371/journal.pone.0042036 (PMC3411616; doi:10.1371/journal.pone.0042036)
Supplement: Table S7 — Temperature and rainfall data for trials in Balm, FL. (DOCX) [file pone.0042036.s007.docx]

**Table S7: Temperature and rainfall data for trials in Balm, FL.**

|  |  | **Fall 2007** | | **Spring 2008** | | **Fall 2008** | | **Spring 2009** | | **Fall 2010** | |
| --- | --- | --- | --- | --- | --- | --- | --- | --- | --- | --- | --- |
|  | **Ave T^a^** | **T^b^** | **% Ave^c^** | **T** | **% Ave** | **T** | **% Ave** | **T** | **% Ave** | **T** | **% Ave** |
| **April** | 68.93 |  |  | 69.66 | 101 |  |  | 70.75 | 103 |  |  |
| **May** | 75.37 |  |  | 77.07 | 102 |  |  | 75.80 | 101 |  |  |
| **June** | 79.12 |  |  | 79.48 | 100 |  |  | 80.62 | 102 |  |  |
| **July** | 79.98 |  |  | 79.74 | 100 |  |  | 80.71 | 101 |  |  |
| **August** | 80.34 | 81.93 | 102 | 80.67 | 100 |  |  |  |  |  |  |
| **September** | 78.80 | 79.26 | 101 |  |  | 80.27 | 102 |  |  | 78.64 | 100 |
| **October** | 73.72 | 76.63 | 104 |  |  | 73.12 | 99 |  |  | 71.91 | 98 |
| **November** | 65.99 | 66.01 | 100 |  |  | 62.83 | 95 |  |  | 65.65 | 99 |
| **December** | 60.61 | 66.30 | 109 |  |  | 64.16 | 106 |  |  | 50.07 | 83 |

**A. Temperatures (degrees F)**

**B. Rainfall (inches)**

|  | **Average** | **Fall 2007** | | **Spring 2008** | | **Fall 2008** | | **Spring 2009** | | **Fall 2010** | |
| --- | --- | --- | --- | --- | --- | --- | --- | --- | --- | --- | --- |
|  | **total rain for month^a^** | **total rain^b^** | **% Ave^c^** | **total rain** | **%Ave** | **total rain** | **%Ave** | **total rain** | **%Ave** | **total rain** | **%Ave** |
| **April** | 1.67 |  |  | 0.03 | 2 |  |  | 0 | 0 |  |  |
| **May** | 2.67 |  |  | 2.81 | 105 |  |  | 6.30 | 236 |  |  |
| **June** | 7.29 |  |  | 5.85 | 80 |  |  | 4.80 | 66 |  |  |
| **July** | 8.85 |  |  | 8.73 | 99 |  |  | 4.39 | 50 |  |  |
| **August** | 7.19 | 5.78 | 80 | 7.63 | 106 |  |  |  |  |  |  |
| **September** | 5.58 | 3.95 | 71 |  |  | 0.64 | 11 |  |  | 3.42 | 61 |
| **October** | 2.26 | 6.42 | 284 |  |  | 1.40 | 62 |  |  | 0.01 | 0 |
| **November** | 1.44 | 0.02 | 1 |  |  | 1.49 | 103 |  |  | 1.24 | 86 |
| **December** | 1.48 | 0.94 | 64 |  |  | 1.36 | 92 |  |  | 0.50 | 34 |

^a^ Average temperatures and average total rainfall for each month were determined by averaging monthly data for the seven years from 2004 to 2010. All weather data was retrieved using the Florida Automated Weather Network (FAWN) weather archive web site.

^b^ Data are reported in each trial period only for months in which trial plants were in the field. Monthly temperature (T) is the average for that month in the given year, and total rain is the total precipitation for the month.

^c^ ‘% Ave’ is the monthly temperature or total rain calculated as a percent of the average values for that month.
